# TNFα Increases *RANKL* Expression via PGE_2_-Induced Activation of NFATc1

**DOI:** 10.3390/ijms18030495

**Published:** 2017-02-24

**Authors:** Hyun-Jung Park, Kyunghwa Baek, Jeong-Hwa Baek, Hyung-Ryong Kim

**Affiliations:** 1 Department of Molecular Genetics, School of Dentistry and Dental Research Institute, Seoul National University, Seoul 08826, Korea; in2010@snu.ac.kr; 2 Department of Pharmacology, College of Dentistry and Research Institute of Oral Science, Gangneung-Wonju National University, Gangwon-do 25457, Korea; daedanhae@gmail.com; 3 Graduate School, DGIST, Daegu 42988, Korea

**Keywords:** TNFα, *RANKL*, PGE_2_, NFATc1, CREB

## Abstract

Tumor necrosis factor α (TNFα) is known to upregulate the expression of receptor activator of NF-κB ligand (*RANKL*). We investigated the role of the calcineurin/nuclear factor of activated T-cells (NFAT) signaling pathway in TNFα-induced *RANKL* expression in C2C12 and primary cultured mouse calvarial cells. TNFα-induced *RANKL* expression was blocked by the calcineurin/NFAT pathway inhibitors. TNFα increased NFAT transcriptional activity and subsequent *RANKL* promoter binding. Mutations in the NFAT-binding element (MT(N)) suppressed TNFα-induced *RANKL* promoter activity. TNFα increased prostaglandin E2 (PGE_2_) production, which in turn enhanced NFAT transcriptional activity and binding to the *RANKL* promoter. MT(N) suppressed PGE_2_-induced *RANKL* promoter activity. TNFα and PGE_2_ increased the expression of *RANKL*, NFAT cytoplasmic-1 (NFATc1), cAMP response element-binding protein (CREB), and cyclooxygenase 2 (COX2); which increment was suppressed by indomethacin, a COX inhibitor. Mutations in the CRE-like element blocked PGE_2_-induced *RANKL* promoter activity. PGE_2_ induced the binding of CREB to the *RANKL* promoter, whereas TNFα increased the binding of both CREB and NFATc1 to this promoter through a process blocked by indomethacin. The PGE_2_ receptor antagonists AH6809 and AH23848 blocked TNFα-induced expression of *RANKL*, NFATc1, and CREB; transcriptional activity of NFAT; and binding of NFATc1 or CREB to the *RANKL* promoter. These results suggest that TNFα-induced *RANKL* expression depends on PGE_2_ production and subsequent transcriptional activation/enhanced binding of NFATc1 and CREB to the *RANKL* promoter.

## 1. Introduction

Receptor activator of nuclear factor-κB ligand (*RANKL*), a critical regulator of osteoclastogenesis, is primarily produced by stromal cells or osteoblasts in bone tissue. *RANKL*, together with colony stimulating factor 1, induces the differentiation of osteoclasts from hematopoietic precursors and stimulates bone resorption of mature osteoclasts [1,2]. Various hormones and cytokines regulate *RANKL* expression in osteoblast or stromal cells through the activation of intracellular signaling pathways, including the cAMP/protein kinase A (PKA), calcineurin/nuclear factor of activated T-cells (NFAT), hedgehog, Wnt/β-catenin, and gp130/STAT pathways [3,4,5,6,7,8].

Tumor necrosis factor α (TNFα) is a multifunctional cytokine that regulates various cellular and biological processes such as cell proliferation, differentiation, apoptosis, immunity, and inflammation [9]. TNFα is known to directly induce bone resorption by activating mature osteoclasts and stimulating the proliferation and differentiation of osteoclast precursors or indirectly by inducing the expression of osteoclastogenic factors in stromal cells or osteoblasts [10,11,12,13].

Several mechanistic pathways have been proposed to determine how TNFα induces the expression of *RANKL* [14,15]. For example, p38 mitogen-activated protein kinase (MAPK) pathway activation mediates TNFα-induced *RANKL* expression and osteoclast differentiation in precursor bone marrow cells [16,17]. Cyclooxygenase (COX)/prostaglandin E (PGE) signaling is also considered a mechanistic pathway by which TNFα induces *RANKL* expression. Prostaglandin E2 (PGE_2_) belongs to the family of prostanoid, autocrine, and paracrine lipid mediators produced by cells following injury or cytokine or growth factor stimulation [18]. PGE_2_ has been described as a potent stimulator of osteoclastic bone resorption in the context of inflammatory diseases such as rheumatoid arthritis and osteomyelitis [19,20,21,22]. PGE_2_ elevates *RANKL* expression in cultured mouse primary osteoblasts [20,23] and human periodontal fibroblasts [24] and is known to bind to any of four G protein-coupled receptors (EP1, EP2, EP3, or EP4) in various target cells [24,25]. COX is a prostaglandin endoperoxide synthase that catalyzes prostaglandin synthesis. Expression of the COX isoform cyclooxygenase 2 (COX2), which is thought to mediate inflammatory events, is rapidly induced by proinflammatory mediators [26,27]. TNFα is known to induce COX2 expression and PGE_2_ production in human gingival fibroblasts via activation of the NFκB pathway [28]. Studies have also reported that TNFα increases *RANKL* expression through the COX2/PGE_2_/EP4/protein kinase A (PKA) signaling pathway [12,19,29].

We previously reported that the cAMP/PKA and calcineurin/NFAT signaling pathways must cooperate to induce parathyroid hormone-related protein (PTHrP)-induced *RANKL* expression in mouse osteoblastic cells [6]. The NFAT family comprises five members: NFAT cytoplasmic-1 (NFATc1) through NFAT5. Calcium signaling pathways dephosphorylate NFATc1 through NFATc4 via the activated calcineurin serine or threonine phosphatase. Dephosphorylated NFATs translocate to the nucleus and regulate the expression of target genes. The calcineurin/NFAT pathway plays an important role in bone resorption, and NFATc1 is a particularly critical transcription factor for osteoclast differentiation [30]. In mice, NFATc1 overexpression in osteoblasts led to increased osteoclast generation and bone resorption [31]. However, the role of the calcineurin/NFAT signaling pathway in TNFα-induced *RANKL* expression remains unexplored.

In the present study, we demonstrated that TNFα induces the transcriptional activation of NFAT via PGE_2_ production and that activation of the calcineurin/NFAT signaling pathway is involved in the TNFα/COX2/PGE_2_-mediated induction of *RANKL* expression.

## 2. Results

### 2.1. Calcineurin/Nuclear Factor of Activated T-Cells (NFAT) Signaling Is Involved in Tumor Necrosis Factor α (TNFα)-Induced Receptor Activator of Nuclear Factor-κB Ligand (RANKL) Expression

**Figure 1:**
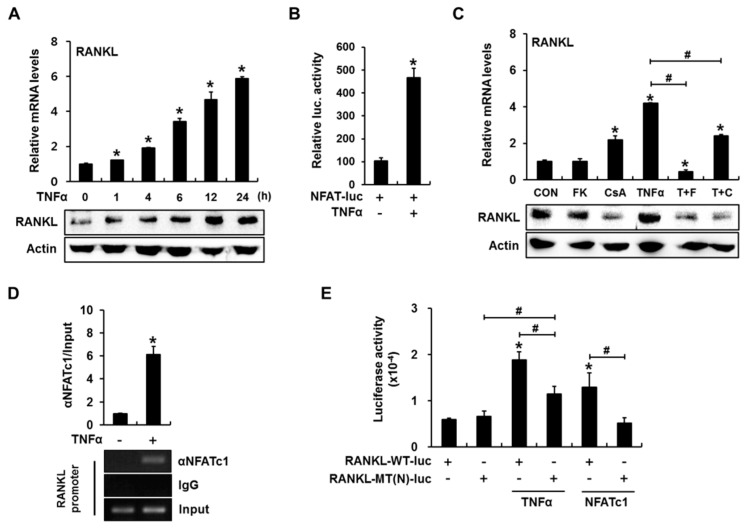
Calcineurin/nuclear factor of activated T-cells (NFAT) activation is involved in tumor necrosis factor α (TNFα)-induced receptor activator of nuclear factor-κB ligand (*RANKL*) expression in C2C12 cells. (**A**) TNFα increased *RANKL* expression in a time-dependent manner. C2C12 cells were incubated in the presence of 10 ng/mL TNFα for the indicated time periods and subjected to quantitative reverse transcription-polymerase chain reaction (RT-PCR) and western blot analyses. Quantitative data are presented as means ± standard deviations (SD); (**B**) TNFα induces NFAT transcriptional activity. C2C12 cells were transfected with a reporter plasmid containing an NFAT response element, exposed to TNFα for 24 h, and subjected to a luciferase assay. Data are presented as firefly luciferase activity levels relative to *Renilla* activity; (**C**) Inhibition of the calcineurin/NFAT pathway blocked TNFα-mediated *RANKL* expression. C2C12 cells pretreated with FK506 (10 μg/mL) or cyclosporin A (10 μg/mL) were treated with TNFα for 24 h and subjected to RT-PCR and western blot analyses; (**D**) TNFα increases NFAT binding to the mouse *RANKL* promoter. C2C12 cells were incubated for 24 h with TNFα, after which a chromatin immunoprecipitation (ChIP) assay was performed using an antibody against NFATc1, with IgG serving as a negative control. The *RANKL* promoter region containing the NFAT binding element was amplified via PCR. Quantitative ChIP data were normalized to the input and are presented as values relative to vehicle-treated control samples (CON) (**E**) TNFα increased *RANKL* promoter–reporter activity in an NFAT binding element-dependent manner. C2C12 cells were transfected with a wild-type (*RANKL*-WT-luc) or NFAT-binding site mutant (*RANKL*-MT(N)-luc) *RANKL* promoter reporter, incubated for 24 h in the presence of TNFα or NFAT overexpression vector, and subjected to a luciferase assay (* *p* < 0.05, compared to control; # *p* < 0.05, compared to the indicated pair).

To confirm the effect of TNFα on *RANKL* expression in C2C12 cells, cells were incubated for 0, 1, 4, 6, 12, and 24 h in the presence of TNFα (10 ng/mL); subsequently, the *RANKL* expression patterns were examined. TNFα clearly upregulated the expression of both *RANKL* mRNA and protein, and TNFα-induced *RANKL* expression reached a peak at 24 h (Figure 1A). Therefore, we chose an incubation period of 24 h for the following experiments.

To determine whether the calcineurin/NFAT pathway is activated by TNFα, we used an NFAT reporter assay with reporter plasmid containing an NFAT response element [32]. TNFα induced an approximately 4.5-fold increase in NFAT reporter activity (Figure 1B). Next, we explored whether the calcineurin/NFAT pathway is involved in TNFα-induced *RANKL* expression. C2C12 cells were treated with TNFα for 24 h in the presence or absence of the calcineurin phosphatase inhibitors FK506 and cyclosporin A (CsA). Although FK506 did not affect the basal *RANKL* expression level, TNFα-induced expression of both *RANKL* mRNA and protein was blocked (Figure 1C). CsA also downregulated both the basal and TNFα-induced *RANKL* protein levels and significantly suppressed TNFα-induced *RANKL* mRNA expression despite increasing the basal *RANKL* mRNA expression (Figure 1C). These results indicate that calcineurin/NFAT pathway activation plays a role in TNFα-mediated *RANKL* induction in C2C12 cells.

Next, a chromatin immunoprecipitation (ChIP) assay was performed to investigate whether TNFα-activated NFAT could directly transactivate the *RANKL* gene by binding to its promoter. The PCR amplification result revealed that TNFα increased the NFATc1 binding to the *RANKL* promoter (Figure 1D). To further confirm that the binding of NFAT to the *RANKL* promoter was functionally important in TNFα-induced *RANKL* expression, luciferase (luc) reporter assays were conducted. *RANKL* promoter–reporter contains −2174 to +1 bp of the mouse *RANKL* gene [4].

When the *RANKL*-WT-luc reporter was used, TNFα induced an approximately three-fold increase in luciferase activity; however, the insertion of mutations into the NFAT binding element (−941 to −936 bp) partially inhibited the TNFα-mediated induction of *RANKL* promoter activity (Figure 1E). NFATc1 overexpression (positive control) resulted in an approximately two-fold induction that was completely blocked by mutations in the NFAT binding element (Figure 1E). These results indicate that TNFα-activated NFATc1 directly binds to and transactivates the *RANKL* promoter.

### 2.2. Inhibition of Calcineurin/NFAT Signaling Partially Reduces TNFα-Induced Prostaglandin E2 (PGE_2_) Production

Previous studies have demonstrated that TNFα increases *RANKL* expression through the COX2/PGE_2_/EP4/PKA signaling pathway [12,19,29] and that NFAT activation enhances both COX2 expression and PGE_2_ production [33,34]. Therefore, we examined whether calcineurin/NFAT signaling pathway activation would mediate TNFα-induced COX2 expression and PGE_2_ production.

**Figure 2:**
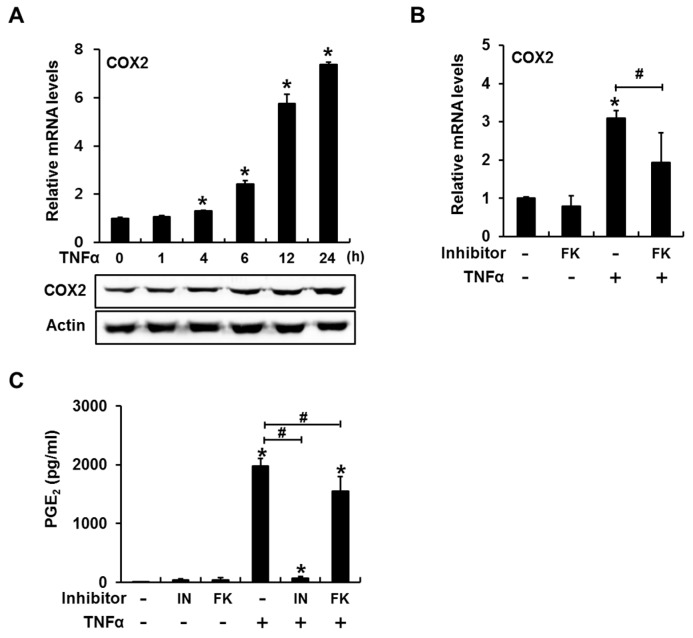
The calcineurin/NFAT signaling pathway mediates TNFα-induced cyclooxygenase 2 (COX2) expression and prostaglandin E2 (PGE_2_) production in C2C12 cells. (**A**) TNFα stimulated COX2 mRNA and protein expression in a time-dependent manner; (**B**) TNFα-mediated COX2 expression decreased following inhibition of the calcineurin/NFAT signaling pathway. C2C12 cells pretreated with the calcineurin phosphatase inhibitor FK506 (10 μg/mL) were incubated in the presence or absence of TNFα for 24 h and subjected to RT-PCR analyses; (**C**) TNFα-induced PGE_2_ production was blocked by indomethacin, a COX inhibitor. Treatment with FK506 slightly suppressed TNFα-induced PGE_2_ production. C2C12 cells were incubated with FK506 or indomethacin (20 μM) in the presence of TNFα for 24 h and subjected to a PGE_2_ parameter assay (* *p* < 0.05, compared to control; # *p* < 0.05).

In C2C12 cells, TNFα treatment increased the expression levels of both COX2 mRNA and protein; these levels peaked at 24 h (Figure 2A). Consistent with this result, TNFα treatment for 24 h strongly induced PGE_2_ production, which was blocked by the COX inhibitor indomethacin (Figure 2C). FK506 did not significantly decrease basal COX2 mRNA expression or PGE_2_ production (Figure 2B,C). However, the addition of FK506 resulted in approximately 40% and 20% decreases in TNFα-induced COX2 mRNA expression and PGE_2_ production, respectively (Figure 2B,C). These results suggest that the calcineurin/NFAT signaling pathway plays a non-critical role in TNFα-induced COX2/PGE_2_ expression.

### 2.3. PGE_2_ Enhances RANKL Promoter Activity in an NFAT Binding Element-Dependent Manner

**Figure 3:**
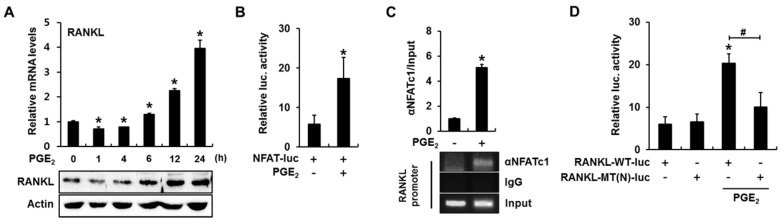
PGE_2_ stimulates *RANKL* expression by activating the calcineurin/NFAT pathway in C2C12 cells. (**A**) PGE_2_ increased *RANKL* expression in a time-dependent manner. C2C12 cells were incubated in the presence of PGE_2_ (50 nM) for the indicated time periods, followed by quantitative RT-PCR and western blot analyses; (**B**) PGE_2_ increased NFAT transcriptional activity. C2C12 cells were transfected with a reporter plasmid containing an NFAT response element, treated with PGE_2_ for 24 h, and subjected to a luciferase assay; (**C**) PGE_2_ induced NFAT binding to the mouse *RANKL* promoter. C2C12 cells were treated with PGE_2_ for 24 h and a ChIP assay was performed; (**D**) PGE_2_ increased *RANKL* promoter-reporter activity in an NFAT binding element-dependent manner. C2C12 cells were transfected with *RANKL*-WT-luc or *RANKL*-MT(N)-luc, incubated for 24 h in the presence of PGE_2_, and subjected to a luciferase assay (* *p* < 0.05, compared to control; # *p* < 0.05).

We next investigated whether calcineurin/NFAT signaling plays a role in PGE_2_-induced *RANKL* expression. When C2C12 cells were incubated in the presence of PGE_2_ (50 nM) for 1, 4, 6, 12, and 24 h, the levels of *RANKL* mRNA and protein were slightly decreased at an early time point, but remarkably increased in a time-dependent manner after 6 h (Figure 3A). PGE_2_ also induced an approximately three-fold increase in NFAT-luc reporter activity (Figure 3B). In addition, a ChIP assay with an NFATc1 antibody revealed that PGE_2_ enhanced NFATc1 binding to the *RANKL* promoter (Figure 3C). Furthermore, PGE_2_ significantly increased luciferase activity when the *RANKL*-WT-luc reporter, but not when the *RANKL*-MT(N)-luc, was used (Figure 3D). These results indicate that PGE_2_ activates the calcineurin/NFAT signaling pathway and that PGE_2_-induced NFAT directly activates *RANKL* transcription by binding to the *RANKL* promoter.

### 2.4. Cyclooxygenase (COX) Inhibitor Blocks TNFα-Induced Binding of NFATc1 and cAMP Response Element-Binding Protein (CREB) to the RANKL Promoter

**Figure 4:**
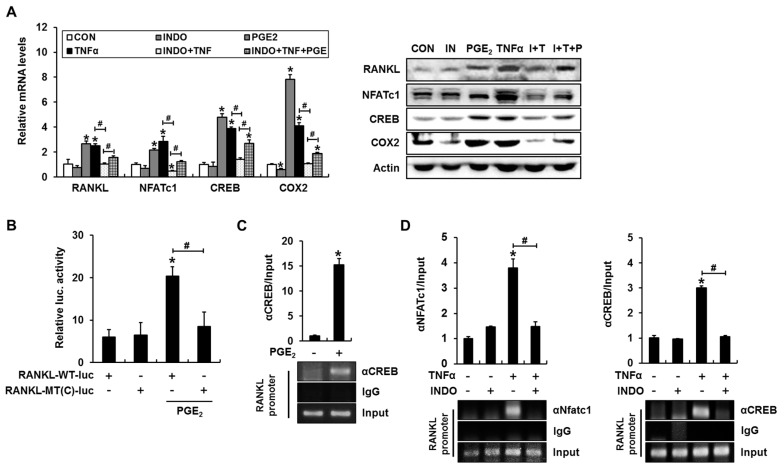
The COX inhibitor indomethacin blocks TNFα-induced binding of NFATc1 and cAMP response element-binding protein (CREB) to the *RANKL* promoter in C2C12 cells. (**A**) PGE_2_ increased the expression of *RANKL*, NFATc1, CREB, and COX2, whereas indomethacin suppressed TNFα-mediated *RANKL*, NFATc1, CREB, and COX2 expression. Treatment with PGE_2_ in the presence of indomethacin and TNFα partially rescued the expression of *RANKL*, NFATc1, CREB, and COX2. C2C12 cells were incubated with the indicated reagents for 24 h and subjected to RT-PCR and western blot analyses; (**B**) PGE_2_ increased *RANKL* promoter–reporter activity in a CREB binding element-dependent manner. C2C12 cells were transfected with *RANKL*-WT-luc or a CREB-binding site mutant (*RANKL*-MT(C)-luc) *RANKL* promoter, incubated for 24 h in the presence of PGE_2_, and subjected to a luciferase assay; (**C**) PGE_2_ induced CREB binding to the mouse *RANKL* promoter. C2C12 cells were incubated for 24 h with PGE_2_ and subjected to a ChIP assay with CREB and control IgG antibodies. The *RANKL* promoter region containing the CREB-binding element was then amplified; (**D**) Indomethacin prevented TNFα-induced NFAT and CREB binding to the mouse *RANKL* promoter. C2C12 cells were incubated for 24 h with the indicated reagents and subjected to a ChIP assay (* *p* < 0.05, compared to control; # *p* < 0.05).

Significant increases in the mRNA and protein expression of *RANKL*, NFATc1, cAMP response element-binding protein (CREB), and COX2 were observed in C2C12 cells incubated with PGE_2_ for 24 h (Figure 4A). TNFα induced similar levels of gene expression, and this process was inhibited by indomethacin (Figure 4A). Further treatment with PGE_2_ in the presence of indomethacin and TNFα partially rescued the expression of *RANKL*, NFATc1, CREB, and COX2. These results suggest that COX/PGE_2_ mediates the TNFα-induced expression of *RANKL*, NFATc1, and CREB.

We previously demonstrated that PTHrP-induced cAMP/PKA signaling promoted NFAT transcriptional activity and that NFAT and CREB cooperate to transactivate the gene encoding *RANKL* in mice [6]. Because our above data demonstrate that PGE_2_ enhances the transcriptional activity of NFAT and its binding to the *RANKL* promoter, we next investigated whether PGE_2_, produced in response to TNFα, would enhance the binding of CREB to the *RANKL* promoter and subsequent transactivation. The *RANKL* promoter–reporter assay demonstrated that mutations inserted in the CRE-like element (−1093 to −1086 bp) prevented PGE_2_ from enhancing reporter activity (Figure 4B). In addition, a ChIP assay with a CREB antibody revealed that PGE_2_ enhanced the binding of CREB to the *RANKL* promoter (Figure 4C). Consistent with these results, TNFα significantly increased the binding of CREB and NFATc1 to the *RANKL* promoter in a process blocked by indomethacin (Figure 4D). These results suggest that TNFα-induced COX/PGE_2_ increases the binding of both CREB and NFAT to the *RANKL* promoter and transcriptional activation of the *RANKL* gene.

### 2.5. The PGE_2_ Receptor Antagonists AH6809 and AH23848 Blocked TNFα-Induced Activation of NFAT and Binding of NFATc1 and CREB to the RANKL Promoter

**Figure 5:**
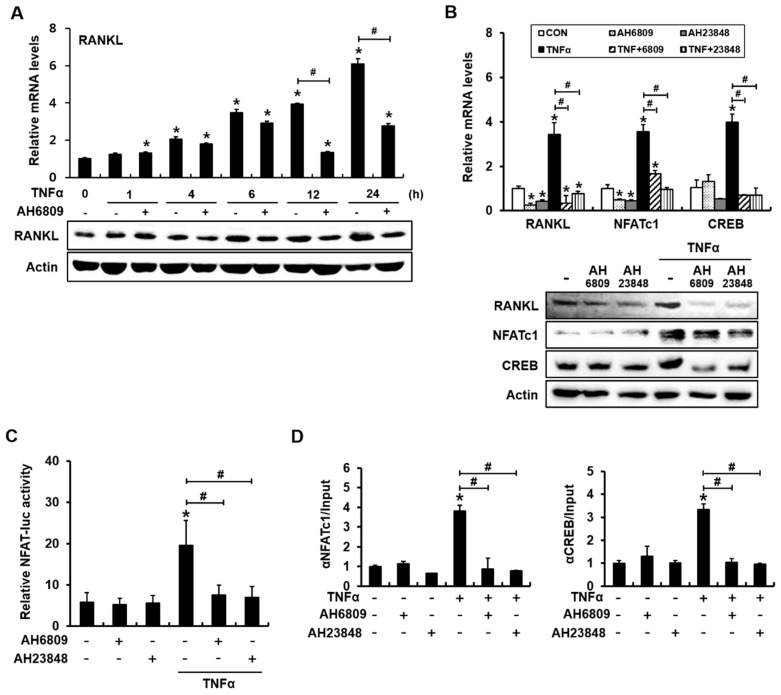
The PGE_2_ receptor antagonists AH6809 and AH23848 suppress the TNFα-induced activation of NFAT and binding of NFATc1 and CREB to the *RANKL* promoter in C2C12 cells. (**A**) PGE_2_ receptor antagonists significantly inhibited TNFα induction of *RANKL* only after incubation for 12 and 24 h; (**B**) AH6809 and AH23848 blocked TNFα-mediated *RANKL*, NFATc1, and CREB expression. AH6809 and AH23848 partially suppressed TNFα-induced NFATc1 protein expression. C2C12 cells were incubated with AH6809 (5 μM) or AH23848 (5 μM) in the presence or absence of TNFα for 24 h; (**C**) PGE_2_ receptor antagonists abolished TNFα-induced NFAT transcriptional activity; (**D**) PGE_2_ receptor antagonists prevented TNFα-induced NFAT and CREB binding to the mouse *RANKL* promoter (* *p* < 0.05, compared to control; # *p* < 0.05).

We next investigated whether PGE_2_ receptor antagonists would inhibit TNFα-induced *RANKL* expression and NFAT activation. AH6809 has the highest affinity for the EP2 receptor, but also acts as a weak antagonist against EP1 and prostaglandin D2 receptor 1 (DP1) receptors in the mouse [35]. AH23848 is a dual antagonist of the EP4 and thromboxane (TP1) receptors [36,37]. C2C12 cells were incubated with AH6809 (5 μM) in the presence of TNFα for 1, 4, 6, 12, and 24 h, and then the levels of *RANKL* mRNA and protein were examined. AH6809 did not significantly suppress TNFα-induced *RANKL* expression at an early time point (6 h or earlier), but inhibited the TNFα-mediated induction of *RANKL* thereafter (Figure 5A). Incubation for 24 h in the presence of AH6809 or AH23848 blocked the TNFα-mediated induction of *RANKL*, NFATc1, and CREB mRNA expression in C2C12 cells (Figure 5B). Compared with NFATc1 mRNA expression, TNFα-induced NFAT protein levels decreased moderately in the presence of AH6809 or AH23848 (Figure 5B). However, TNFα-induced NFAT transcriptional activity was abolished by the addition of AH6809 or AH23848 (Figure 5C). Furthermore, ChIP assays with antibodies to NFATc1 or CREB demonstrated that PGE_2_ receptor antagonists suppressed TNFα-induced binding of NFATc1 or CREB to the *RANKL* promoter (Figure 5D). These results indicate that TNFα-induced *RANKL* expression depends on the production of PGE_2_, which subsequently binds to EP2 or EP4 receptor and enhances the binding and transcriptional activity of NFATc1 and CREB in the mouse *RANKL* promoter.

### 2.6. TNFα Enhances RANKL Expression in a PGE_2_ Production- and NFAT Activation-Dependent Manner in Primary Cultured Mouse Calvarial Cells

**Figure 6:**
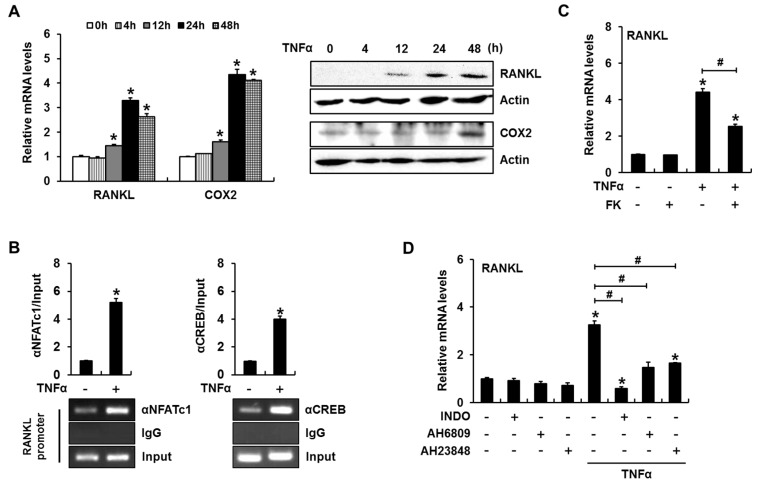
PGE_2_ production and NFAT activation are necessary for TNFα-induced *RANKL* expression in primary cultured mouse calvarial cells. (**A**) TNFα enhanced the expression of *RANKL* and COX2. Calvarial cells were incubated in the presence of TNFα for the indicated time periods and subjected to RT-PCR and western blot analyses; (**B**) ChIP assays revealed that TNFα increased NFATc1 and CREB binding to their cognate binding sites in the mouse *RANKL* promoter; (**C**) A calcineurin/NFAT signaling inhibitor suppressed TNFα-induced *RANKL* expression. Calvarial cells were incubated for 24 h in the presence of FK506 and TNFα; (**D**) Treatment with a COX2 inhibitor or PGE_2_ receptor antagonists suppressed TNFα-induced *RANKL* expression. (* *p* < 0.05, compared to control; # *p* < 0.05).

We next confirmed the roles of PGE_2_ and calcineurin/NFAT signaling with regard to TNFα-regulated *RANKL* expression in primary cultured mouse calvarial (MC) cells. Consistent with our observations in C2C12 cells, TNFα induced both *RANKL* and COX2 mRNA and protein expression (Figure 6A). PCR amplification of the DNA region containing the NFAT binding element or CRE-like region revealed that TNFα enhanced the binding of NFATc1 or CREB to the *RANKL* promoter (Figure 6B). FK506-mediated inhibition of NFAT activation significantly reduced TNFα-induced *RANKL* expression (Figure 6C). Indomethacin, AH6809, and AH23848 also inhibited TNFα-induced *RANKL* mRNA expression (Figure 6D). These results further suggest that PGE_2_ mediates TNFα-induced *RANKL* expression through a process that involves enhanced binding of NFAT and CREB to the *RANKL* promoter.

## 3. Discussion

In the present study, we demonstrated that TNFα-induced *RANKL* expression involves both PGE_2_-induced activation of the calcineurin/NFAT pathway and subsequent binding of NFATc1 to the *RANKL* promoter. The following results of our study support the involvement of PGE_2_ and the calcineurin/NFAT signaling pathway in TNFα-induced *RANKL* expression: (i) TNFα stimulated COX2 expression and PGE_2_ production; (ii) a COX inhibitor or EP antagonists blocked TNFα-induced *RANKL* expression; (iii) TNFα-induced *RANKL* expression was significantly downregulated by inhibition of the calcineurin/NFAT pathway; (iv) TNFα enhanced the transcriptional activity of NFATc1 and binding of NFATc1 to the *RANKL* promoter; (v) TNFα-induced *RANKL* promoter–reporter activity was attenuated by mutations in the NFAT binding element; and (vi) a COX inhibitor or EP antagonists blocked TNFα-enhanced transcriptional activity and binding of NFAT to the *RANKL* promoter.

It has been well demonstrated that *RANKL* expression increases with TNFα treatment in a dose-dependent manner and 10 ng/mL of TNFα was used as a representative dose in several studies [38,39,40,41,42,43]. Therefore, we adopted a concentration of 10 ng/mL in the present study.

The ability of TNFα to stimulate PGE_2_ production via COX2 has been well demonstrated [28,44,45,46], and PGE_2_ is known to activate the cAMP/PKA signaling pathway in target cells [19]. Of the PGE_2_ receptor subtypes, EP4 has the highest expression in fibroblasts. Compared to control mice, EP4-deficient mice exhibited reduced inflammatory responses in a collagen-induced arthritis model, fewer osteoclasts, and reduced *RANKL* expression in osteoblast cells [20,47,48,49]. These reports suggest that EP4 is critical to the stimulation of bone resorption. Another report demonstrated that in MC osteoblastic cells, PGE_2_-induced cAMP production and *RANKL* expression are mainly mediated by EP4, although EP4 and EP2 must cooperate to ensure a full response to PGE_2_ [23]. In the present study, either an EP2 or EP4 antagonist completely blocked TNFα-induced *RANKL* expression. In addition, both antagonists abolished TNFα-induced NFAT reporter activity and the binding of NFATc1 and CREB to the *RANKL* promoter in C2C12 cells. In primary cultured MC cells, both antagonists had similar partial inhibitory effects on TNFα-induced *RANKL* expression. These results suggest that both EP2 and EP4 are involved in PGE_2_-induced *RANKL* expression. However, it is not clear why the responses of primary MC cells and C2C12 cells to EP antagonists differ.

In the present study, we did not examine the receptor subtypes involved in TNFα-induction of COX2 and PGE_2_ production. TNFα regulates cellular activities via the activation of either of two receptors: TNF receptor 1 (TNFR1) and TNFR2. TNFR1 is expressed ubiquitously, whereas TNFR2 expression is restricted to specific cell types, including immune cells and neurons [50]. Previous reports have demonstrated that TNFα induces COX2 expression and PGE_2_ production via TNFR1 in synovial fibroblasts [51,52]. Furthermore, induction of *RANKL* by TNFα was abolished in TNFR1-deficient mouse gingival epithelial cells [29]. These studies suggest that the activation of TNFR1 contributes to TNFα-induced activation of COX2 and production of PGE_2_ in C2C12 and mouse calvarial cells.

Although the PGE_2_-induced cAMP/PKA signaling is known to induce *RANKL* expression, the effector molecules downstream of PKA have not yet been clearly elucidated. Previously, PKA was shown to target CREB and thus increase the extent of CREB binding to distal enhancers of *RANKL* [53]. Assuming that PGE_2_/PKA signaling mediates TNFα-induced *RANKL* expression, we propose NFAT to be another transcription factor that acts downstream of PGE_2_ to induce *RANKL* transcription. Similar to a previous report in which CREB and NFATc1 cooperation was necessary to induce *RANKL* transcription via the PTHrP/cAMP/PKA pathway [6], TNFα-induced PGE_2_ enhanced the binding of CREB and NFATc1 to the mouse *RANKL* promoter, and mutations in either NFAT-binding element or the CRE-like element blocked PGE_2_-induced *RANKL* promoter activity. In addition, indomethacin and EP antagonists prevented TNFα-induced binding of NFATc1 and CREB to the *RANKL* promoter. These data support a role for NFATc1 as another mediator linking PGE_2_/PKA signaling to *RANKL* transcription.

A rich body of literature demonstrates that NFAT activation directly regulates COX expression and PGE_2_ production. Notably, NFAT activation regulates COX2-encoding genes in human colon carcinoma [33]. In this study, COX2 expression and PGE_2_ production were induced in response to NFAT stimuli and blocked when CsA or FK506 was used to inhibit calcineurin phosphatase activity. Lipopolysaccharide-induced NFAT activation was shown to regulate PGE_2_ synthesis in dendritic cells [34], and NFAT was found to induce COX2 transcription in human glioblastoma cells [54] and to regulate constitutive COX2 expression in the renal medulla [55]. Furthermore, PTH was shown to induce *COX2* transcription through cross-talk between the cAMP/PKA and calcineurin/NFAT signaling pathways in murine osteoblastic cells [56]. Therefore, we determined whether inhibition of the calcineurin/NFAT pathway would suppress COX2-induced PGE_2_ production. FK506 partially suppressed TNFα-induced COX2 expression and PGE_2_ production. However, functional PGE_2_ inhibition by EP antagonists completely abolished the TNFα-induced transcriptional activity of NFAT, indicating that TNFα-induced NFAT activation is PGE_2_-dependent and subsequently contributes to the further induction of COX2 expression.

In the present study, TNFα-induced COX2 expression was obvious only after incubation for 4 h, although TNFα-induced *RANKL* expression was significant even after 1 h-incubation in C2C12 cells. In addition, PGE_2_ antagonist AH6809 did not significantly suppress TNFα-induced *RANKL* expression at an early time point (6 h or earlier). Given the data demonstrating the difference between the early vs. late response, it is suggested that there is a PGE_2_-independent pathway in *RANKL* induction of TNFα in the immediate response. Considering the continuing effect of TNFα and PGE_2_ on *RANKL* induction after 12 h, PGE_2_ seems to mediate the slow, delayed, long-term *RANKL* expression by TNFα, which is more likely to be associated with pathologic conditions such as inflammatory bone resorption.

In conclusion, the present study demonstrates that TNFα-induced *RANKL* expression depends on PGE_2_ production and subsequent transcriptional activation, as well as on the enhanced binding of NFATc1 and CREB to the *RANKL* promoter in mouse osteoblastic cells.

## 4. Materials and Methods

### 4.1. Reagents and Antibodies

Recombinant human TNFα was purchased from R&D Systems (Minneapolis, MN, USA). FK506, cyclosporin A, SB203580, H89, PGE_2_, and indomethacin were purchased from Sigma (St. Louis, MO, USA). AH6809 and AH23848 were purchased from Cayman Chemical (Ann Arbor, MI, USA). *RANKL* antibody was purchased from R&D Systems. Antibodies to NFATc1, COX2, and β-actin and horseradish peroxidase (HRP)-conjugated secondary antibodies were obtained from Santa Cruz Biotechnology (Dallas, TX, USA). CREB antibody was purchased from Cell Signaling Technology (Danvers, MA, USA).

### 4.2. Cell Culture

C2C12 cells, a murine mesenchymal cell line that can be differentiated into osteoblasts [57], were maintained in Dulbecco’s modified Eagle’s medium (DMEM) supplemented with 10% fetal bovine serum (FBS), 100 U/mL of penicillin, and 100 μg/mL of streptomycin.

MC cells were isolated from the frontal and parietal bones of neonatal ICR mice, as previously described [58]. Before preparation of MC cells, animals were weaned in mouse gang cages following Institutional Animal Care and Use Committee policies. The animal study was reviewed and approved by the Special Committee on Animal Welfare, Seoul National University, Seoul, Republic of Korea (approval no. SNU-20140228-1-5). MC cells were cultured in α-minimum essential medium (α-MEM) supplemented with 10% FBS, 100 U/mL of penicillin, and 100 μg/mL of streptomycin. DMEM, α-MEM, and FBS were obtained from Hyclone (Walkersville, MD, USA).

### 4.3. Plasmid Construction

Construction of the NFATc1 expression vector and a NFAT reporter plasmid has been described in previous publications [32,59]. Construction of a *RANKL* promoter-luciferase reporter plasmid (*RANKL*-WT: −2174 to +1 bp of the mouse *RANKL* promoter) and a function-defective mutant reporter containing mutations in the NFAT-binding site (*RANKL*-MT-N; GGAAAA⟶GC*tt*AA) [4] or in the CRE-like element (*RANKL*-MT-C; TGAGGTCA⟶TGAGG*agg*) have been described in previous publications [6,60,61].

### 4.4. Reverse Transcription-Polymerase Chain Reaction

Quantitative reverse transcription-polymerase chain reaction (RT-PCR) was performed as described previously [6]. PCR primers with the following sequences were synthesized by Cosmogenetech (Seoul, Korea): *RANKL*-forward (F) 5′-CAG AAG ATG GCA CTC ACT GCA-3′, *RANKL*-reverse (R) 5′-CAC CAT CGC TTT CTC TGC TCT-3′; NFATc1-F 5′-AAT AAC ATG CGA GCC ATC ATC-3′, NFATc1-R 5′-TCA CCC TGG TGT TCT TCC TC-3′; CREB-F 5′-AGC TGC CAC TCA GCC GGG TA-3′, CREB-R 5′-TGG TGC TCG TGG GTG CTG TG-3′; COX2-F 5′-CCA GCA CTT CAC CCA TCA GTT-3′; COX2-R 5′-ACC CAG GTC CTC GCT TAT GA-3′; glyceraldehyde 3-phosphate dehydrogenase (GAPDH)-F 5′-TCA ATG ACA ACT TTG TCA AGC-3′; and GAPDH-R 5′-CCA GGG TTT CTT ACT CCT TGG-3′. GAPDH was used as a reference to normalize each sample for quantification.

### 4.5. Western Blot Analysis

The western blot analysis was performed as described previously [6]. Immune complexes were visualized using EZ-Western Lumi Pico (Daeillab Service Co., Seoul, Korea), and chemiluminescence was detected using a MicroChemi device (DNR, Jerusalem, Israel).

### 4.6. Chromatin Immunoprecipitation Assay

The chromatin immunoprecipitation (ChIP) assay was performed as described previously [4]. Cross-linked DNA fragments were subjected to pre-clearing with blocked protein G agarose, and immunoprecipitation was performed with an NFATc1 or CREB antibody or species-matched control IgG. After purification, DNA sequences were analyzed by PCR amplification of the mouse *RANKL* promoter region encompassing either the NFAT-binding element (amplified region: −1070 to −858 bp) or CRE-like element (amplified region: −1147 to −967 bp). PCR primers with the following sequences were used: NFAT-binding element forward, 5′-GCA AGC TCC AGG CCA GCC TAG-3′ and reverse, 5′-CCA ATA AGA CGG CTC AGC TG-3′; CRE-like element forward, 5′-AGG AGG CAG AGA TGG CAG AG-3′ and reverse, 5′-ACA CGC GCG CGC GCA AAT A-3′.

### 4.7. Luciferase Reporter Assay

The luciferase reporter assay was performed as described previously [6]. In order to normalize transfection efficiency, *Renilla* luciferase plasmid was co-transfected with *RANKL* promoter reporters.

### 4.8. PGE_2_ Assay

Cells were incubated for 24 h in medium supplemented with the indicated reagents, after which supernatants were collected. The concentration of PGE_2_ in the supernatant was determined using Parameter PGE_2_ (R&D Systems) according to the manufacturer’s instructions.

### 4.9. Statistical Analysis

Statistical significance was determined using Student’s *t*-test. For the multiple comparisons, one way ANOVA was performed. When significant main effects were detected, post hoc analyses were conducted with the least squares means error test. Differences were considered significant at *p* < 0.05. Data were analyzed by using the SAS program (version 9.1; SAS Institute, Cary, NC, USA).
